# Analysis of Key Issues on GNSS-R Soil Moisture Retrieval Based on Different Antenna Patterns

**DOI:** 10.3390/s18082498

**Published:** 2018-08-01

**Authors:** Fei Li, Xuefeng Peng, Xiuwan Chen, Maolin Liu, Liwen Xu

**Affiliations:** Institute of Remote Sensing and GIS, Peking University, No. 5 Yiheyuan Road, Haidian District, Beijing 100871, China; xfpeng@pku.edu.cn (X.P.); xwchen@pku.edu.cn (X.C.); maolin@pku.edu.cn (M.L.); liwenxu22@126.com (L.X.)

**Keywords:** GNSS-R, antenna pattern, soil moisture, spatial resolution, detection depth

## Abstract

GNSS-R (Global Navigation Satellite System-Reflectometry) has been demonstrated to be a new and powerful tool to sense soil moisture in recent years. Multi-antenna pattern and single-antenna pattern have been proposed regarding how to receive and process reflected signals. Great efforts have been made concerning ground-based and air-borne observations. Meanwhile, a number of satellite-based missions have also been implemented. For the in-depth study of soil moisture remote sensing by the technique of GNSS-R, regardless of the extraction methods of the reflected signals or the types of the observation platform, three key issues have to be determined: The specular reflection point, the spatial resolution and the detection depth in the soil. However, in current literatures, there are no comprehensive explanations of the above three key issues. This paper conducts theoretical analysis and formula derivation, aiming to systematically and quantitatively determine the extent of soil moisture being detected in three dimensions from the above-mentioned aspects. To further explain how the three factors behave in the specific application, the results of two application scenarios are shown: (1) a ground-based GPS measurement in Marshall, Colorado, US from the Plate Boundary Observatory, corresponding to single-antenna pattern. The relative location of the specular reflection points, the average area of the First Fresnel Ellipse Clusters and the sensing depth of the time-series soil moisture are analyzed, and (2) an aviation experiment conducted in Zhengzhou to retrieve soil moisture content, corresponding to the multi-antenna pattern. The spatial distribution of soil moisture estimation with a certain resolution based on the flight tracks and the relevant sensing depth are manifested. For remote sensing using GNSS reflected signals, BeiDou is different from GPS mainly in the carrier frequency. Therefore, the results of this study can provide references for China’s future development of the BeiDou-R technique.

## 1. Introduction

An important branch of the global navigation satellite system (GNSS) is GNSS reflectometry (GNSS-R), which has emerged in recent years. It utilizes the code-modulated L-band microwave signal that has long-term stability and high spatiotemporal resolution provided by navigation satellites, combining the advantages of both active radar and passive radiometers [1]. GNSS-R has demonstrated great potential in applications such as sea surface height, sea surface wind field, sea ice detection, vegetation status, soil moisture, and snow depth [2,3,4,5,6]. Currently, various ground-based, air-based and even satellite-based observation experiments are widely conducted worldwide to actively promote its relevant applications. For example, the TDS-1 satellite launched by the UK in 2014 was equipped with SGR-ReSI sensors to facilitate GNSS-R measurements and is currently used in studies for the retrieval of soil moisture [7,8]. The National Aeronautics and Space Administration (NASA) launched the CYGNSS Satellite Constellation in October 2016 [9], and the European Space Agency (ESA) deployed the GEROS-ISS [10] and the PARIS IoD Program [11]. Although the Chinese GNSS-R study focuses on ground-based and air-based methods, China has included a satellite-based GNSS-R observation program in its “Thirteenth Five-Year Plan” and “Fourteenth Five-Year Plan”. In addition, the radar transmitter originally installed in the Soil Moisture Active Passive (SMAP) malfunctioned in July 2015. The receiver is currently switched to receive GPS L2C frequency, and it also becomes a satellite-borne GNSS-R observation [12,13]. In the future, more abundant GNSS-R data will be used to measure parameters of the Earth’s surface.

Soil moisture is an important parameter in the fields of agriculture, hydrology, meteorology and ecology. In recent years, scientists have conducted extensive investigations in the field of soil moisture remote sensing via land- and air-based GNSS-R. Masters et al. calculated the reflection coefficient by calculating the maximum value of the reflected power and the delayed power waveform of the direct signal, and then obtained the dielectric constant through the bistatic radar equation, and finally estimated the soil moisture through an empirical model. This is also known as the GNSS-ICF method [14]. In 2002, NASA added GPS bistatic radar measurements in the Soil Moisture Experiment 2002 (SMEX02) for the first time and confirmed that the intensity of the reflected signal has a spatiotemporal correlation with soil moisture [15]. ESA conducted ground- and air-based GNSS-R tests in Italy in March to September of 2009 and in July and August of 2011, respectively, to acquire the direct signal of right-handed circular polarization (RHCP) and the reflected signal of RHCP/left-handed circular polarization (LHCP); the results showed that the ratio of the direct signal of RHCP to the reflected signal of LHCP can be used as a reliable measurement for soil moisture [16,17]. Multiple ground- and air-based GNSS-R measurements on soil moisture have been performed by the Polytechnic University of Catalonia, Spain, from 2013 to 2015; in these studies, multipolar measurements of direct and reflected GNSS signals were conducted, and the effect of the instrument parameters on the calculation of the reflection coefficient was examined [18,19,20]. Chew et al. used the GPS L2C band reflection signal data provided by the SMAP satellite to identify the surface freezing and fusion status [21]. In terms of the interference of direct and reflected GNSS signals, Larson developed the technique of GPS-multipath reflectometry (GPS-MR) for soil moisture measurement using the GPS signal-to-noise ratio (SNR) data from the crustal deformation monitoring network [22]. Rodriguez-Alvarez et al. developed the Interference Pattern Technique (IPT) for measuring soil moisture using a special receiver and a vertically polarized antenna. The University of Colorado carried out an analysis and a simulation of relevant interference parameters and soil moisture, and obtained real-time results of soil moisture estimation based on GPS SNR data, and published the results on the relevant websites of PBO GPS station network data [23]. At the same time, with the development of other GNSS systems and UAV remote sensing platforms, there has been some UAV-based GNSS-R soil moisture retrieval studies [24]. Roussel et al. explored the feasibility of soil moisture estimation based on GLONASS SNR data and studied the effect of satellite elevation angle on the accuracy of soil moisture inversion [25]. Yang Lei et al. proposed soil moisture inversion method based on Support Vector Regression (SVRM) from the BeiDou Geostationary Orbit (GEO) satellite reflection signal [26].

Based on the receiving and processing modes of the reflected signals, Wan et al. categorized the GNSS-R remote sensing observation into two modes: single-antenna observation pattern and multiple-antenna observation pattern [27]. For soil moisture observation, the ultimate goal of the two observation patterns is to extend the scientific findings to business applications. Whether using the single-antenna pattern that separates the reflected components from interference signals, the multi-antenna pattern that measures the reflected signal separately, ground-based GNSS-R observation, or air- or satellite-based measurement, three common key issues need to be addressed for practical applications: The geographical location of the signal reflection, the earth surface range of the reflected signal, and the soil depth represented by the carried information, i.e., the specular reflection point of GNSS-R, the spatial resolution, and the detection depth. These three key factors that are involved in soil moisture remote sensing with GNSS-R have been sporadically investigated. Currently, comprehensive, systematic, and quantitative analyses are still lacking. In this study, first, from the theoretical perspective, we present the interpretation, the general mathematical descriptions, and the considerations for some special observation conditions. Then, we introduce the applications for the ground- and air-based observations in the single-antenna pattern and in the multi-antenna pattern using the Plate Boundary Observatory (PBO) soil moisture product from the United States and the aviation observation data for farmland around Zhengzhou City, Henan Province, as examples. Thus, this study provides implications for the research and business applications of soil moisture remote sensing via GNSS-R.

## 2. Theoretical Analysis of Key Issues on Soil Moisture Retrieval

To promote the research and business application processes of soil moisture remote sensing via GNSS-R, the soil detection range of the reflected GNSS signal must be clarified from the perspective of three-dimensional space. A specular reflection point can help determine the geographic location, while the spatial resolution and detection depth help determine the volume of the detected soil cube. Because the recording of soil moisture information occurs when GNSS signals are reflected on the Earth’s surface, the following three problems are present in both single-antenna and multi-antenna GNSS-R remote sensing patterns.

### 2.1. Specular Reflection Point of GNSS Signals

In the geometric relationship between the direct signal and the reflected signal, the point that has the shortest path among the transmitter, surface, and receiver is called the specular reflection point, at which the incidence angle of the GNSS signal is equal to the signal’s reflection angle. Thus, the specular reflection point serves as an important reference point for GNSS-R measurement and modeling. In ground-based observations, the antenna is generally 2 to 3 m in height, and the observation surface can be regarded as a plane. Then, the specular reflection point can be calculated using the satellite’s elevation angle and azimuth angle based on simple geometric relationships. In air- and satellite-based observations, the observation platform is high above the ground, and the observed surface must be viewed as a curved one.

**Figure 1:**
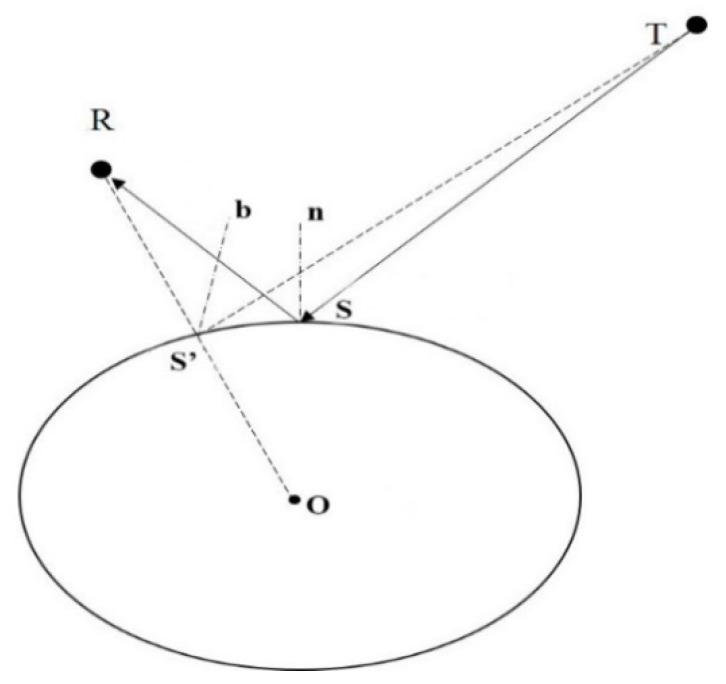
Geometry of the GNSS-R observation system.

The WGS84 ellipsoid corresponding to the WGS84 coordinate system used by GPS broadcast ephemeris has been used as a simple and effective geometric approximation of the Earth’s surface. Figure 1 shows the geometry of the GNSS-R observation system. When the location of the transmitter (T) and receiver (R) are known, the specular reflection point (S) can be determined using an iterative method. In this method, the location where the line connecting the center of the earth (O) and the receiver (R) intersects the ground is used as the initial point (S’). Then, the ground point S’ is continuously moved toward the bisector of the angle formed by S’R and S’T. When the mobile measurement of S’ is smaller than the set threshold value, S’ is determined as the specular reflection point (S), where the bisector of the angle formed by SR and ST is the normal line of Point S that passes through the curved surface.

**Figure 2:**
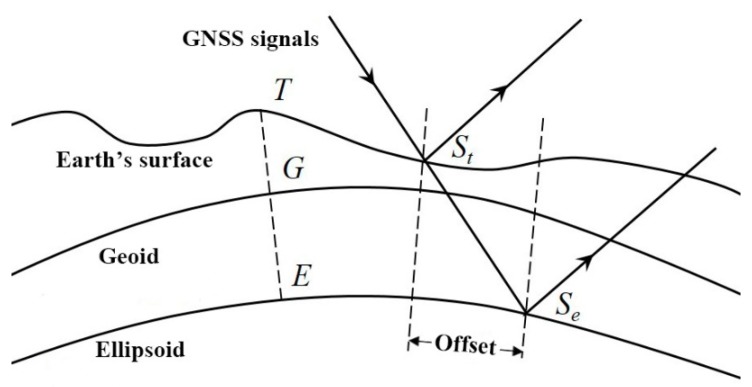
Effect of geoid and terrain height on the specular reflection point.

However, the approximation of the Earth’s surface using the WGS84 ellipsoid leads to a certain error, and geoid and terrain height must also be taken into account. The geoid is an isotropic gravitational surface that corresponds to the global average sea level and is more in line with the natural shape of the Earth. The heights of an ellipsoid and of a geoid can differ by tens of meters. The height measured relative to the geoid is called altitude, which is a common height measurement used in many topographic maps and digital data sets [28]. Figure 2 shows the influence of geoid and terrain height on the specular reflection point, where the ellipsoidal height (ET) is the sum of the geoid height (EG) and altitude (GT). Considering only the ellipsoid surface while neglecting the geoid and terrain height will cause a certain deflection in determining the specular reflection point. As shown in Figure 2, Se is the specular reflection point when only the ellipsoid is taken into account, and St is the specular reflection point when both the geoid and the terrain elevation are considered. Assuming the height difference between the Earth’s true surface and the WGS84 ellipsoid is Δ*h*, then the latitude and longitude deflections of the specular reflection point (Δ*lat*, Δ*lon*) are as follows [29]:

(1) $$\Delta lat=\frac{\Delta h\cdot \cot\gamma \cdot \cos\varphi }{R_{e}},\;\Delta lon=\frac{\Delta h\cdot \cot\gamma \cdot \sin\varphi }{R_{e}\cdot \cos y}\;$$

where *γ* and *φ* are the elevation angle and azimuth of the GNSS satellite relative to the specular reflection point, respectively, and *y* is the latitude at the specular reflection point. Then, the radius (*Re*) of the ellipsoid can be calculated from the major semi-axis (*ae*) and the minor semi-axis (*be*):

(2) $$R_{e}^{2}=\frac{b_{e}^{2}}{1+\left(b_{e}^{2}/a_{e}^{2}\right)\cdot \sin y}$$

**Figure 3:**
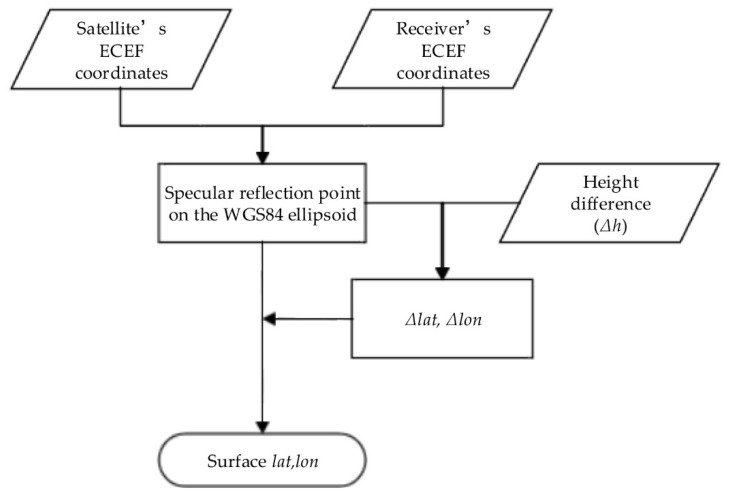
Calculation procedure of the specular reflection point in GNSS-R.

Based on the XYZ coordinates in the Earth-centered Earth-fixed (ECEF) coordinate system of the GNSS satellite and the receiver, the latitude and longitude of the specular reflection point on the WGS84 ellipsoid can be obtained. Then, based on the height difference (Δ*h*) between the Earth’s true surface and the WGS84 ellipsoid surface, the changes in latitude and longitude can be obtained. These changes are then added to the latitude and longitude of the WGS84 ellipsoid to obtain the latitude and longitude of the specular reflection point on the Earth’s true surface. The overall procedure is shown in Figure 3.

### 2.2. Spatial Resolution of Remote Sensing Via GNSS-R

**Figure 4:**
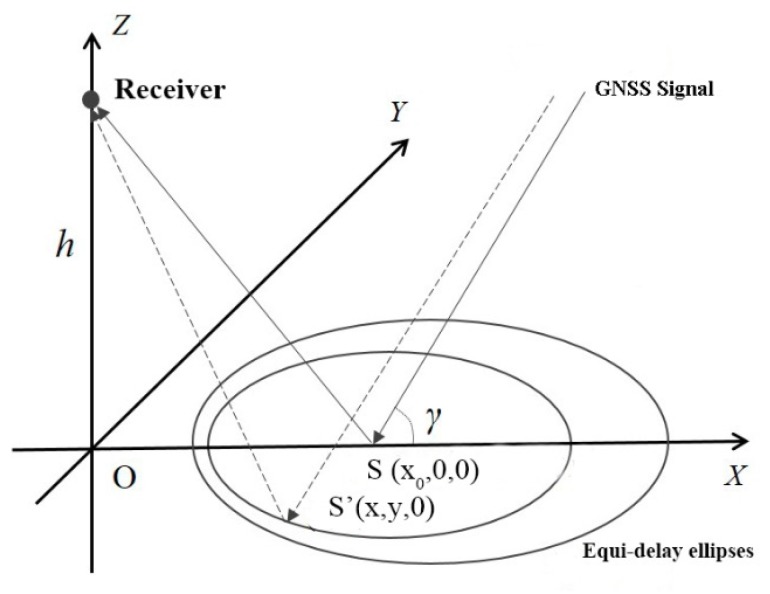
Schematic diagram of equi-delay ellipses of the reflected GNSS signal.

In the GNSS-R system, the ground is set as the X-Y plane, and the direction of the receiver pointing to the zenith is set as the Z axis to establish the right-handed rectangular coordinate system, as shown in Figure 4. The GNSS signal travels the shortest path by passing the specular reflection point (x_0_,0,0) in a grazing angle of *γ* and then being reflected to reach the receiver at the height of *h*. Assuming that the signal that is reflected at the point (x,y,0) is also received and that the difference between the travel distance of the signal passing through the point (x,y,0) and that of the signal passing through the specular reflection point is *δ*, then *δ* satisfies the following formula [30]:

(3) $$\delta =\sqrt{x^{2}+y^{2}+h^{2}}-x\cos\gamma -h\sin\gamma$$

The trajectory of the point (x,y) on the X-Y plane is an ellipse, and its major semi-axis and minor semi-axis *b* are:

(4) $$a=\frac{\sqrt{\delta^{2}+2\delta h\sin\gamma }}{\sin^{2}\gamma },\;b=\frac{\sqrt{\delta^{2}+2\delta h\sin\gamma }}{\sin\gamma }$$

The coordinates of the center of the ellipse are:

$$\left(\frac{\delta +h\sin\gamma }{\sin\gamma \tan\gamma },\;0\right)$$

**Figure 5:**
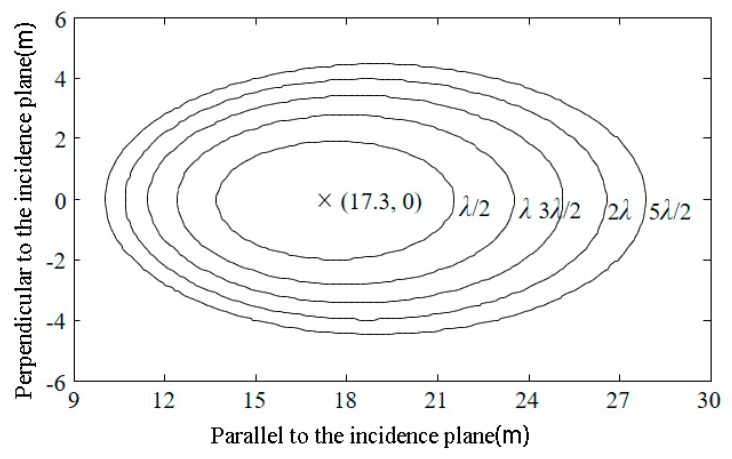
The shape of Equi-delay ellipses at a receiver height of 10 m and a grazing angle of 30°.

The GNSS-R configuration can be considered as a bistatic radar system where the size and the shape of the scattering scene, called Glistening Zone (GZ), depend on system configuration, i.e., receiver altitude and observation angle, receiver antenna and surface roughness [31]. The signal at the receiver, mainly coming from the SP, is correlated with a set of local replicas of the GNSS code shifted in time delay and Doppler frequency. Hence, the received signal is mapped in the Delay-Doppler domain to generate the so-called Delay-Doppler Map (DDM). In case of airborne or fixed platform receiver configuration, the Doppler effect can be neglected. For the height of the receiver is 10 m, the grazing angle of the GNSS signal is 30°, and *δ* is set to *λ*/2, *λ*, 3*λ*/2, 2*λ*, and 5*λ*/2 (for the L1 band of GPS, *λ* = 0.19 m); the size and shape of the equi-delay ellipses are shown in Figure 5. In this figure, the coordinates of the projection of the receiver on the Earth’s surface is (0,0), and the specular reflection point location is labeled “×”. The corresponding *δ* to the equi-delay ellipses increases sequentially from the inside outward.

If the relationship between the ground surface and the incident wavelength is in accordance with the Fraunhofer criterion, i.e., the surface is approximately smooth to the L-band microwave, then a typical approximation is to replace *δ* with *λ*/2 so that the location and size of the ellipse can be determined. In this case, the ellipse is called the first Fresnel zone. Because it is the “first reflection zone” of the bistatic radar, the size of the area can be viewed as the spatial resolution of remote sensing via GNSS-R [32].

(5) $$S=\pi ab=\frac{\pi \left(\delta^{2}+2\delta h\sin\gamma \right)}{\sin^{3}\gamma }=\frac{\pi \lambda \left(\lambda +4h\sin\gamma \right)}{4\sin^{3}\gamma }$$

Formula (5) can be regarded as the spatial pixel size of soil moisture remote sensing via GNSS-R. Taking the L1 (1575.42 MHz) band of GPS as an example, the elevation angles of the GNSS satellite are 5, 10, 20, 30, 50 and 70°, and the spatial resolutions calculated using Formula (5) are shown in Table 1. For ground-based observation platforms, the height of the antenna phase center from the ground is generally controlled between 1.5 and 2.5 m to exclude interference from the surrounding environment when the signal is at a low satellite elevation angle. Air-based observation platforms generally use manned aircrafts or drones to carry relevant loads, and the height is about 1 km.

Because the spatial resolution of the airborne GNSS-R at an elevation angle of 5° is only 3151 m^2^, the effect of the Earth’s curvature is negligible in the estimation, and the first Fresnel zone still can be approximated as a plane. Analysis of Table 1 shows that when the platform height is fixed, the spatial resolution and the satellite elevation angle show a decreasing relationship; when the satellite elevation angle is fixed, the spatial resolution increases with the height of the platform. At the same time, the GNSS-R technology is not based on the observation of points in a very small space, and the selection of different elevation angles and platform heights can meet the needs of different spatial resolutions.

The above discussion is based on the premise of a smooth ground, where the area of the first Fresnel zone is precisely the size of the GZ. However, surface state cannot be considered homogeneous at medium to large scales especially in case of a spaceborne receiver configuration, where the conventional GNSS-R DDM is not enough to include nonhomogeneous areas. Schiavulli proposed an inversion scheme based on the two-dimensional (2-D) truncated singular-value decomposition (TSVD) to reconstruct the NRCS field from noisy DDMs (both thermal and speckle noise are accounted for) related to a simulated marine scenario, which includes different nonhomogeneous areas and discussed an almost uniform spatial resolution that characterizes the resolution of the reconstructed image [33].

For airborne receivers or receivers with lower altitudes, since their altitude is much smaller than the height of the GNSS satellites, the effect of the curvature of the earth can be ignored. The GZ can be regarded as the cut of the earth passing the specular reflection point and mainly concentrated in the first equi-delay ellipse, even the surface is rough. In order to obtain surface roughness information from the waveform, the GZ must contain several equal-pitch annular zones (three or more), and when the receiver height increases, the flash zone can contain multiple equi-delays, which describe the spatial resolution of a large surface.

In the case that the ground surface has a certain gradient, the cosine value of the slope needs to be taken into account on top of the area of the orthographic projection. It needs to be pointed out that the frequency of the 1561.098 MHz) of the BDS satellite is very close to that of the L1 band of the GPS, the spatial resolutions of GNSS-R remote sensing under the same conditions are essentially identical. Whether the observation platform is ground based, air based or satellite based, the spatial resolution of soil moisture remote sensing via GNSS-R can be evaluated at between the conventional satellite remote sensing and the resolution of the ground-site measurement, which complements the advantages of various methods in the observational scale.

### 2.3. GNSS-R Soil Detection Depth of GNSS Signals

**Figure 6:**
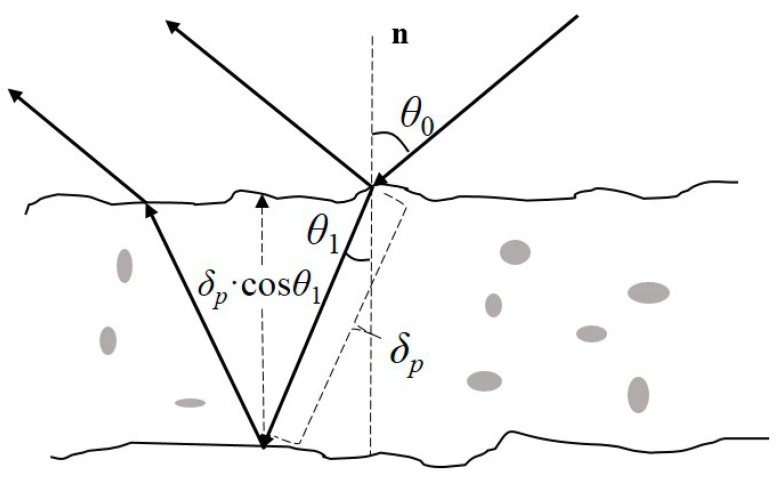
Propagation of an electromagnetic wave and its penetration distance in soil.

For electromagnetic waves with a given frequency, the soil dielectric constant is generally a function of soil moisture, bulk density, soil composition, temperature and salinity, with soil moisture exerting the greatest effect [34]. The propagation distance of an electromagnetic wave in the medium until the electromagnetic wave energy is attenuated to 1/*e* is referred to as the penetration distance (*δ p* in Figure 6). The attenuation of electromagnetic wave energy is affected by soil moisture, which changes the signal’s penetration distance.

If the soil is not very dry, the penetration distance of an electromagnetic wave is in the range of 0.1*λ* to *λ*, where *λ* corresponds to the volumetric soil moisture mv ≈ 0.04 cm^3^/cm^3^ and while 0.1*λ* corresponds to very moist soil. In Figure 6, the detection depth is *δ p*·cos *θ*_1_, where *θ*_1_ is the refraction angle of the signal with an incident angle of *θ*_0_ in the soil. For soils with a uniform dielectric constant, 63% of the energy of the signal radiating in the direction with an angle of *θ*_0_ to the normal line after interacting with the soil are from the detected depth. Thus, the estimation based on the reflected signal is primarily the soil moisture information in the range of the detection depth.

According to electromagnetic wave theory, the formula of plane wave propagation in a homogeneous lossy medium is as follows [35]:

(6) $$\vec{E_{}}\left(z\right)=\vec{E_{0}}\left(z\right)\cdot exp\left(-j\omega \sqrt{\mu \varepsilon_{c}}\cdot z\right)$$

where $\vec{E_{}}$ is the electric field vector; *z* indicates the propagation direction; and *ω*, *μ*, $\varepsilon_{c}$, and *j* are the angular frequency, permeability, complex permittivity, and imaginary unit, respectively. Then, the propagation constant ($j\omega \sqrt{\mu \varepsilon_{c}}$) can be expressed as:

(7) $$j\omega \sqrt{\mu \varepsilon_{c}}=j\omega \sqrt{\mu \varepsilon_{0}\cdot \varepsilon_{r}}=j\cdot \frac{2\pi }{\lambda }\sqrt{\varepsilon_{r}}=\alpha +j\cdot \beta$$

where $\varepsilon_{r}$ is the relative complex permittivity and can be expressed as $\varepsilon_{r}=\varepsilon_{r}^{\prime }-j\cdot \varepsilon_{r}^{\prime \prime }$; $\varepsilon_{0}$, *α*, and *β* are the complex permittivity of air, the attenuation constant, and the phase constant, respectively. Then, the attenuation constant α can be expressed as:

(8) $$\alpha =\frac{2\pi }{\lambda }\left|Im\left(\sqrt{\varepsilon_{r}}\right)\right|=\frac{\pi \varepsilon_{r}^{\prime \prime }}{\lambda \sqrt{\varepsilon_{r}^{\prime }}}$$

For the microwave band, the imaginary part of the complex permittivity of soil is much smaller than the real part. Therefore, $\sqrt{\varepsilon_{r}^{\prime }-j\cdot \varepsilon_{r}^{\prime \prime }}$ can be performed with Taylor series expansion, where Im(⋅) represents the solution of the imaginary part of the complex number in the brackets.

Assuming that the power of the electromagnetic wave is *p*(0) at the soil-air interface, the power of the electromagnetic wave after traveling in the soil a distance of z satisfies Formula (9) [36]:

(9) $$p(z)=p(0)\cdot e^{-2\alpha z}$$

When *p*(z)/*p*(0) = 1/e, z = *δ p*:

(10) $$\delta_{p}=\frac{1}{2\alpha }=\frac{\lambda \sqrt{\varepsilon_{r}^{\prime }}}{2\pi \varepsilon_{r}^{\prime \prime }}$$

The relationship between the incident angle *θ*_0_ and the angle of refraction *θ*_1_ can be determined through the refractive index of the microwave in the soil ($n_{1}$):

(11) $$\frac{\sin\theta_{0}}{\sin\theta_{1}}=n_{1}=\sqrt{\varepsilon_{r}^{\prime }}$$

When the frequency of the electromagnetic wave is 1.4 GHz, Hallikainen et al. presented the calculation formulas for the real and imaginary parts of the complex permittivity of soil [37]:

(12) $$\{\begin{array}{c} \varepsilon =\varepsilon^{\prime }-j\varepsilon^{\prime \prime }\\ \varepsilon^{\prime }=1.15*{\left[1+\frac{\rho_{b}}{\rho_{s}}\left(\varepsilon_{s}^{\alpha }\right)+m_{v}^{\beta^{1}}\varepsilon_{fw}^{\prime \alpha }-m_{v}\right]}^{1/\alpha }-0.68\\ \varepsilon^{\prime \prime }={\left[m_{v}^{\beta^{\prime \prime }}\varepsilon_{fw}^{\prime \prime \alpha }\right]}^{1/\alpha } \end{array}$$

where *ρ_b_* is the solid soil volume density; *ρ_s_* is the specific gravity value of solid soil (2.66 g/cm^3^); the α value is 0.65, which is the empirical constant; *ε’_fw_* and *ε’’_fw_* are the permittivity of free water for the real part and imaginary part respectively; *β′* and *β″* are empirical constants related to soil type, and the expression is [38]:

(13) $$\{\begin{array}{c} \beta^{\prime }=1.2748-0.519S-0.152C\\ \beta^{\prime \prime }=1.3380-0.603S-0.166C \end{array}$$

where S and C are sandy and clay contents respectively.

**Figure 7:**
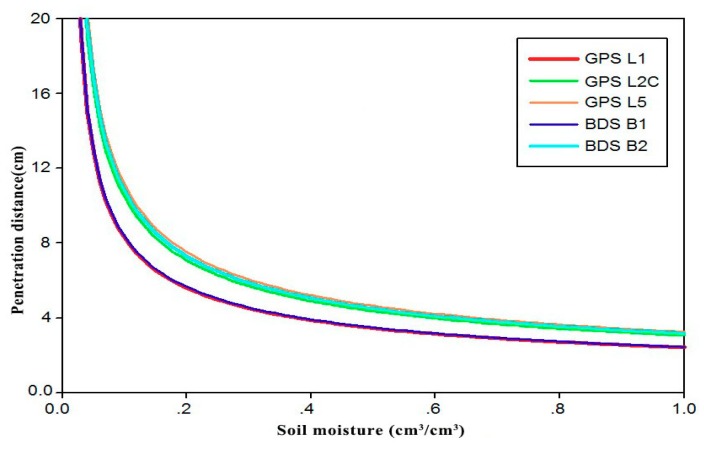
Dependence of the penetration distance on soil moisture for different frequency bands.

Figure 7 shows the depth of penetration of BDS and GPS in different frequency bands when S and C are 50% respectively, considering different soil moisture conditions except for extremely dry soil, of which the penetration depth is between 0 and 20 cm. When the soil moisture increases from 0 to 0.2 cm^3^/cm^3^, the depth of penetration rapidly decreased to within 8 cm. When the soil moisture changes from 0.2 to 1.0 cm^3^/cm^3^, the penetration distance slowly declined. When the soil moisture is at higher values (VSM > 0.5 cm^3^/cm^3^), the probe depth was reduced to within 5 cm. Therefore, during the process of collecting soil moisture data, two depth data of 2.5 and 7.5 cm are generally selected for collection. Further analysis shows that for BDS and GPS and different frequency bands, when the soil moisture is the same, the higher the carrier frequency of the signal is, the smaller the penetration distance is. This is related to the fact that the higher the frequency of the electromagnetic wave is, the faster it decays in the soil. The BDS B1 band is equal to the wavelength of the GPS L1 band (~19 cm), so the penetration distance values of the two are relatively consistent. Similarly, the range of variation between GPS L2C, L5, and BDS B2 bands is small (24~25.5 cm). Therefore, the difference in penetration distance is not significant.

**Figure 8:**
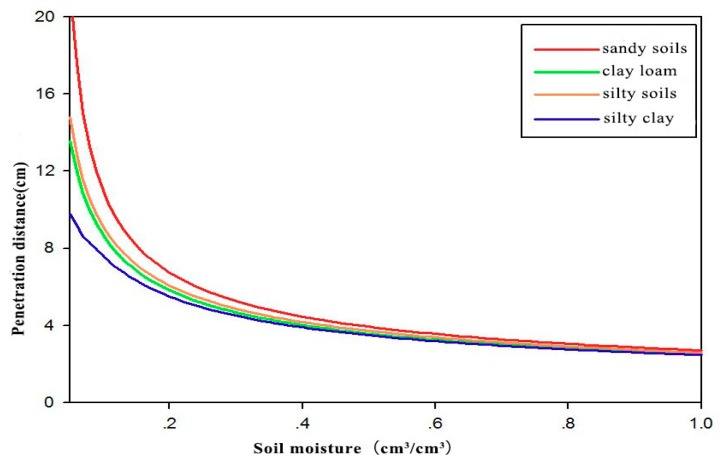
Dependence of the penetration distance of the GPS L1 band signal on soil moisture for different soil types.

Similarly, for the example of the L1 band of GPS, Figure 8 shows the changes in the signal penetration distances in four typical soil types (sandy soils, clay loam, silty soils, and silty clay) with soil moisture. This result indicates that for all soil types, the penetration distance decreases as the soil moisture increases. When the soil is dry (VSM < 0.2 cm^3^/cm^3^), the soil composition has a greater influence on the depth of penetration. With the increase of soil moisture, the soil composition has little effect on penetration distance. When the soil moisture exceeds 0.2 cm^3^/cm^3^, the penetration distance will be shorter than 6 cm.

In addition, after estimating the soil moisture through GNSS-R, the penetration distance of the signal in the soil can be determined based on the signal frequency and the soil type; it is then multiplied by cos *θ*_1_ to obtain the detection depth. The GNSS-R soil moisture remote sensing result is an average value of soil moisture within the detection depth range.

## 3. Results and Discussion of Key Issues in Application Scenarios

### 3.1. Ground-Based Observation in Single-Antenna Pattern

The PBO Program of the United States investigates the changes in the three-dimensional stress field of the tectonically active crust of the West Pacific and North American plates, and it currently has 1100 continuously operating GPS stations. Based on the SNR data recorded by these GPS stations, the University of Colorado launched a soil moisture product (http://xenon.colorado.edu/portal/). In this study, Station P041 in Colorado (39.9495° N, 105.1943° W) is used as an example to address the three key issues mentioned above.

**Figure 9:**
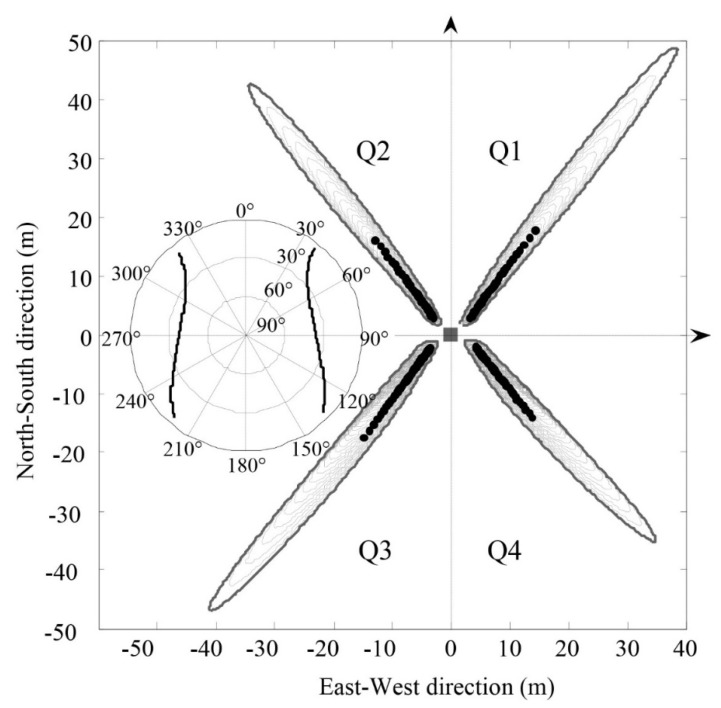
The specular reflection point and the first Fresnel zone of the PRN 1 satellite signal at Station P041 on 1 January 2012, where the sub-map is the sky map of the PRN 1 satellite.

The antenna height at Station P041 is 2 m, and the PRN 1 satellite data from 1 January 2012, which were acquired with an elevation angle range of 5~25° and a sampling interval of 1 min, were chosen. Given the known antenna height, the specular reflection point can be determined based on the elevation and azimuth angles. Then, the first Fresnel zone is determined based on Formula (3), as shown in Figure 9. In this figure, the origin of the coordinates is the location of the GPS observation station, and the sub-map is the sky map of the PRN 1 satellite on 1 January 2012. On this date, a total of two satellites rose and set, and the elevation angles of the four sections were in line with the range of 5~25°. The resultant four specular reflection point trajectories were distributed in four quadrants.

In the ground-based single-antenna observation pattern, the surface soil moisture information is directly extracted from the interference waveforms of direct and reflected signals [19], and the interference requires changes to the satellite’s elevation angle. Thus, the soil moisture estimated from an interference pattern represents the soil moisture information in the surface range passed through by the first Fresnel zone during the time period. The area covered by each ellipse cluster in Figure 8 is the spatial resolution of the corresponding soil moisture estimate, and the average area of the ellipse clusters in four quadrants is 205 m^2^. According to Formula (5), the area of the first Fresnel zone is determined by the wavelength of the microwave, the antenna height, and the grazing angle, while the area of the ellipse cluster also depends on the azimuth angle and the range of the satellite’s elevation angle.

**Figure 10:**
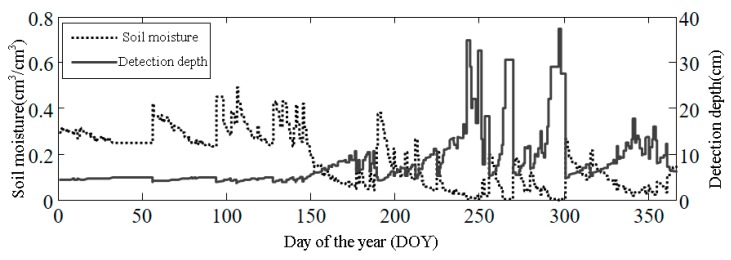
Daily soil moisture throughout the year 2012 and the detection depth of the GNSS signal at Station P041.

Based on the soil moisture estimates from the SNR data using the elevation angle range of 5~25° [19] and setting the elevation angle of the GNSS signals to 15°, the daily soil moisture data of the entire year of 2012 at Station P041 is derived from the PBO product. The corresponding detection depth can be calculated using Formulas (10) and (11), as shown in Figure 10. This figure shows that when the soil moisture is in the range of 0.2~0.4 cm^3^/cm^3^, the detection depth is approximately 5 cm. When the soil is very dry, the detection depth can be deeper than 20 cm. Larson et al. used the wire net that covers the first Fresnel zone to determine the detection depth of the GNSS signal in the soil and found that when the soil moisture was 0.1 cm^3^/cm^3^, the detection depth of the L2-band signal was 5 ± 1 cm, in contrast to 7.6~9.2 cm under the same conditions using the model described in Section 2.3. This model assumes that the soil moisture is uniform, but in reality, the soil moisture changes with soil depth, which should be the main cause of error. In addition, different soil types also lead to variations in the signal’s penetration distance. Therefore, the model described in Section 2.3 can be used to determine the relative depth of signal detection.

### 3.2. Air-Based Observation in Multi-Antenna Pattern

**Figure 11:**
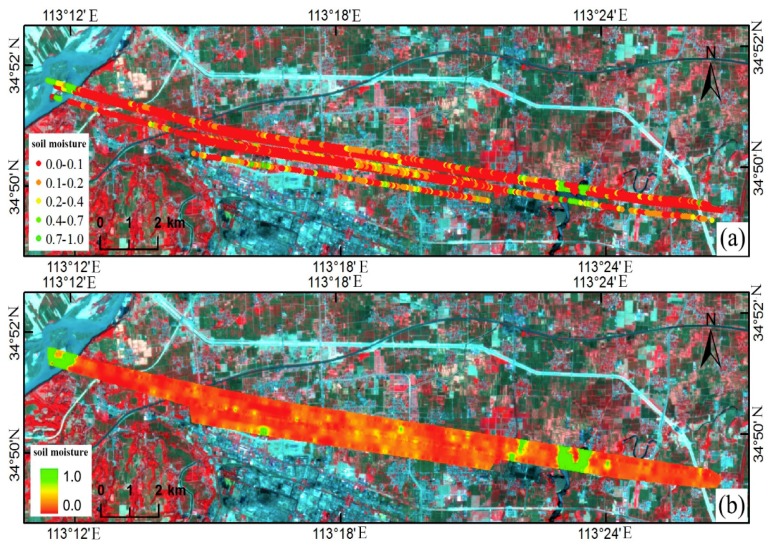
Soil moisture estimation result of an aviation experiment with GNSS-R over Zhengzhou. (**a**) Distribution of soil moisture at the specular reflection point; (**b**) soil water grid data (at a resolution of 20 × 20 m).

Next, using the air-based GNSS-R experiment conducted in Zhengzhou on 30 May 2014 as an example, the common key issues in the application of air-based observation in multi-antenna pattern are further analyzed. The experiment was conducted using the Yun-12 aircraft-borne GNSS-R load developed by the National Space Science Center of the Chinese Academy of Sciences to observe the regional farmland soil moisture and the water surface height of the Yellow River. The flight height was approximately 500 m, and the grazing angles of the signals from four satellites (PRN 18, 24, 21 and 22) during the flight were rather large. Specifically, the left-handed reflected signal was used to estimate soil moisture. The spatial location of the specular reflection point was determined according to the procedure shown in Figure 3. Then, the corresponding soil moisture estimate was superimposed on the remote sensing image for analysis, as shown in Figure 11a. In this figure, the base map is the GF-1 WFV pseudo-color composite image (transit date: 26 May 2014) with a resolution of 16 m, and the specular reflection points with different colors indicate different soil moisture ranges, as shown in the figure legends. In this study, we focused on the key issues common to GNSS-R soil moisture remote sensing; for an analysis of the soil moisture estimation accuracy, please refer to the literature [27].

The average grazing angle of the signals of the four selected satellites was 58°. According to Formula (5), the resolution pixel of this air-based GNSS-R test was 420 m^2^ on average. Using the inverse distance weighted interpolation method on the observation area, the soil moisture value for a spatial resolution of 20 × 20 m was obtained, as shown in Figure 11b. In this figure, as the color changes from red to green, the soil moisture estimates increase. The northwestern part of the study area is the Yellow River, and there is a pond in the east. The color boundaries in the grid image accurately depict the boundaries of the water and the bank, indicating that the GNSS-R technology can also be applied to land cover classification. The remaining parts of the map are mainly red and yellow, which is related to the fact that no precipitation occurred two weeks before the test and that the soil throughout the study area was thus rather dry.

**Figure 12:**
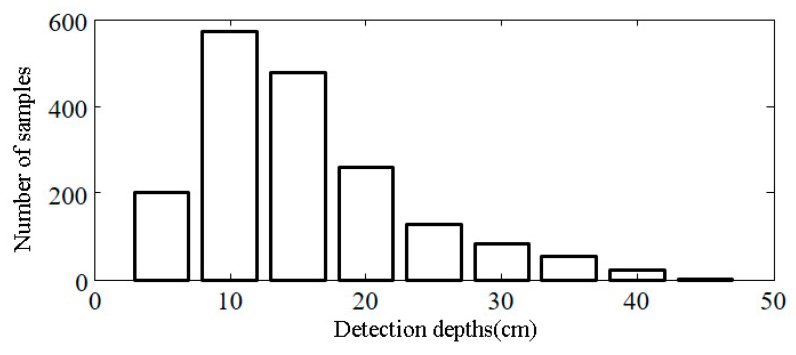
Soil moisture detection depths histogram generated from an aviation experiment with GNSS-R in Zhengzhou.

Using formulas in Section 2.3, the detection depth of each soil moisture estimate can be calculated, and the detection depths histogram of all the sampling points is shown in Figure 12. This figure shows that the detection depths of most sampling points were 10~20 cm. Because the soil was rather dry throughout the study area during the aviation experiment, the soil moisture estimates of most sampling points were lower than 0.1 cm^3^/cm^3^. This again indicates that the GNSS signals detection depths is closely related to soil moisture. This conclusion can provide reference information to verify the accuracy of airborne GNSS-R. Specifically, when laying out the soil moisture meters to collect the measurement data, different measurement depths can be set according to the characteristics of the uneven spatial distribution of soil moisture. Thus, a more targeted accuracy evaluation of the GNSS-R soil moisture estimates can be performed.

## 4. Conclusions

Since the reflected signals from GNSS have begun to be used to obtain surface parameters through remote sensing, the GNSS-R technology has been gradually extended from sea surface remote sensing applications to land surface remote sensing applications. As an important branch of land surface remote sensing, soil moisture remote sensing via GNSS-R has formed two observation patterns (single-antenna and multi-antenna patterns) along with their respective research methods. Currently, multiple low-orbit satellites equipped with a GNSS-R device have been launched to facilitate various in-flight observation programs worldwide. To improve the technical maturity of soil moisture remote sensing via GNSS-R and to promote its business applications, it is necessary to evaluate the accuracy of the soil moisture estimation. The keys to this evaluation are determining the geographic location of the specular reflection point, the spatial resolution and the soil detection depth. A feasible operational basis can be provided for evaluating the accuracy of GNSS-R soil moisture retrieval by clarifying these three key issues.

In this study, an iterative method for determining the location of the specular reflection point is presented, and the effect of geoid and terrain height on the location of the specular reflection point is analyzed. Based on the discussion of the geometric characteristics of equi-delay ellipses of the reflected signal of GNSS, the concept of the resolution of GNSS remote sensing is introduced, and its mathematical formulas are presented. For smooth and rough ground, the spatial resolution algorithm is different. In reference to microwave remote sensing theory, the calculation formula of the penetration distance of GNSS signals in the soil is also presented, while the concepts of detection depth and penetration distance are distinguished. Using ground-based observation via the single-antenna pattern and air-based observation via the multi-antenna pattern as examples, this study analyzed three common key issues from the perspective of practical applications. For the single-antenna pattern, to provide a reference for the product use and accuracy evaluation, this study used the published PBO soil moisture product to present the relative location of the specular reflection point, the surface range and area of the reflected GPS signal, and the estimated detection depth represented by soil moisture in the time series. The interference signals which contains direct signals and ground reflection signals can be obtained from the signal noise ratio (SNR) data. After the analysis of the interference waveform, the direct signal can be removed and the sinusoidal waveform can be used for calculation. For the multi-antenna pattern, the structure can be regarded as a bistatic radar. In this study, using the air-based preliminary studies conducted, we quantitatively described the spatial distribution, the spatial resolution, and the detection depth of soil moisture via GNSS-R.

Moreover, in terms of surface parameter remote sensing, the signal of the BeiDou satellite mainly differs from the GPS in the frequency of the band. Therefore, the analyses of the above common key problems are also applicable to remote sensing via the reflected signal of the BeiDou satellite. A quantitative reference was provided for the application of remote sensing using the reflected signal from the BeiDou satellite via the single-antenna pattern. Furthermore, future development will likely shift from air-based preliminary studies to satellite-based observation. This study provide references for the satellite orbit and load design of future satellite-based observation as well as the soil moisture estimation model and products.

## Figures and Tables

**Table 1:** Spatial resolutions (m^2^) of GNSS-R in the B1 band of GPS at different observation platform heights and satellite elevation angles.

Observation Platform	Satellite Elevation Angle (°)					
	5	10	20	30	50	70
Ground-based (2 m)	16.3	7.7	3.7	2.3	1.4	1.2
Air-based (1 km)	3151	1581	803	500	316	265
